# A scoping review of stroke registers in Sub-Saharan Africa

**DOI:** 10.1177/17474930241262936

**Published:** 2024-07-31

**Authors:** Daniel Youkee, Mamadu Baldeh, Anthony Rudd, Marina Soley-Bori, Charles DA Wolfe, Gibrilla F Deen, Iain J Marshall

**Affiliations:** 1 School of Life course & Population Health Sciences, King’s College London, London, UK; 2 College of Medicine and Allied Health Sciences, The University of Sierra Leone, Freetown, Sierra Leone

**Keywords:** Stroke, register, registry, Sierra Leone, Africa, Sub-Saharan Africa

## Abstract

**Background:**

Stroke registers are recommended as a key priority by the Lancet Neurology World Stroke Organization Commission for Stroke, 2023, and the African Stroke Leaders’ Summit, 2022.

**Aims:**

This scoping review aims to map where stroke registers have been implemented in Sub-Saharan Africa (SSA). The article then compares and critiques the methods and definitions used and summarizes key results from the registers. The scoping review searched EMBASE, MEDLINE, and CABI Global Health databases and included all studies with a prospective longitudinal design in SSA, where adult acute stroke was the primary condition studied. Articles were screened against inclusion and exclusion criteria independently by two authors.

**Summary:**

We identified 42 unique stroke registers from 48 individual studies. The registers were located in 19 countries, with 19 from East Africa, 15 West Africa, 6 Central Africa, and 2 from Southern Africa. Cumulatively, the registers recruited 12,345 participants with stroke, the median number of participants was 183 (interquartile range (IQR): 121–312), and the range was 50–1018. Only one study was a population-based register, and 41 were hospital-based registers. Of the hospital-based registers, 29 were single site, 10 were conducted at two sites, and 2 at three sites. Twenty-three (54.7%) of the registers were located in the capital city of their respective country, and only one of the hospital-based registers was in a self-described rural area. Length of recruitment ranged from 4 months to 6 years; the median length of recruitment was 12 months. Methodology and definitions were heterogenous between the registers. Only seven (19.4%) registers referenced the WHO STEPwise approach to implementing stroke registers. Twenty-seven (64.3%) registers used the WHO definition of stroke. The mean neuroimaging rate was 84%, and ranged from 0% to 100%. Stroke severity was measured using the National Institute of Health Stroke Scale (NIHSS) in 22 (52.4%) registers, four registers used the Glasgow Coma Scale (GCS), two registers used the miniNIHSS, one used the Scandinavian Stroke Scale, one modified Rankin Scale (mRS), and 11 registers did not report a stroke severity measure. Seventeen (40.5%) registers used the mRS to measure function, six registers used Barthel Index alone, and three registers used both mRS and Barthel Index. Only two registers included a quality-of-life measure, the EQ-5D. Eight registers included a quality-of-care measure, and 26 (61.9%) registers recorded socioeconomic status or a socioeconomic status proxy, most frequently educational attainment.

**Conclusions:**

This scoping review found high heterogeneity of methods and definitions used by stroke registers, with low uptake of the WHO stepwise method of stroke surveillance. A drive to standardize methodology would improve the comparability of stroke data in SSA. The shared use of educational attainment by registers in our review may enable future meta-analyses of inequities in stroke in SSA. Incorporating health-related quality-of-life measures, such as EQ-5D, into stroke registers should be encouraged, bringing a patient perspective, and allow the estimation of quality-adjusted life years lost to stroke. Agreement on a standardized register methodology or further promotion and uptake of the WHO stepwise method is essential to produce comparable data to improve stroke prevention and care.

## Introduction

Stroke registers have been implemented in some countries since the 1980s and are considered a key priority by the Lancet Neurology World Stroke Organization Commission for Stroke^1^ and the African Stroke Leaders Summit in 2022. Fewer stroke registries have been reported in low-income countries,^2^ such as those in Sub-Saharan Africa (SSA), where estimates suggest disease incidence is increasing.^3^ Stroke registers are a type of clinical register,

an organized system that uses observational study methods to collect uniform data (clinical and other) to evaluate specified outcomes for a population defined by a particular disease, condition, or exposure, and that serves one or more predetermined scientific, clinical, or policy purposes.^1^

Stroke registers often have multiple purposes including but not exclusive to

to observe the course of disease; to understand variations in treatment and outcomes; to examine factors that influence prognosis and quality of life; to describe care patterns, including appropriateness of care and disparities in the delivery of care; to assess effectiveness; to monitor safety and harm; and to measure quality of care.^4^

Since the 1980s, there are pertinent examples of the use of national and international stroke registers to generate evidence on stroke epidemiology and care. The WHO Multinational Monitoring of Trends and Determinants in Cardiovascular Disease (MONICA) international stroke register aimed to assess and compare the magnitude of stroke in the community, the social and clinical profile of stroke patients, and the natural history of stroke in different populations.^5^ Hypothesizing that the characteristics of stroke, stroke types, and disease progression may vary across different groups and geographical regions, they compared registries from 16 European and 2 Asian countries and found a more than threefold difference in stroke mortality rates between countries.^6^ The South London Stroke register has demonstrated changes in stroke incidence between different age and ethnic groups.^7^ The Riks-stroke, a national stroke registry in Sweden, has described how baseline characteristics of stroke patients, such as age and risk factors, have changed over 13 years.^8^ Stroke registers have supported primary prevention programs to tackle risk factors and improve management over time.^9,10^ While the effectiveness of stroke units on health outcomes has been demonstrated in randomized controlled trials,^11^ registers have demonstrated these improvements in health outcomes and quality of care in the real world.^12,13^ Registers have been used to evaluate cost-effectiveness of new interventions such as thrombectomy,^14^ and to evaluate major health system change such as centralizing stroke services.^15^ Stroke registers have been used to monitor quality of care^16^ and examine disparities in quality of care.^17^ While the outputs of stroke registers are clearly recorded, there is less evidence of whether stroke registers drive improvements in health services^18^ or merely observe change.^19^

One potential of stroke registers is to measure stroke risk, incidence, and outcomes over time and compare these between countries or regions, thereby highlighting inequities and supporting the targeting of public health and health system interventions. A comparison of data between registers requires common methodology and standardized definitions and instruments. A systematic review of national stroke registries found that “a large number of variables were collected but definitions, numerators, and denominators for similar variables were inconsistent across registries, making international comparisons difficult.”^16^ It has been questioned whether the WHO (MONICA)-reported variations in incidence and outcomes after stroke are due to real differences in disease burden or due to differences in case ascertainment and methodology between sites.^20^ Some authors claim that “Worldwide variation in the impact of stroke may be attributed to methodological and case ascertainment differences.”^21^ As a collaborative attempt to provide improved comparable data on stroke, the WHO recommended a standard method for developing stroke registers in low- and middle-income countries in 2005.^22^ The method comprises three steps: Step one involved establishment of hospital-based stroke registers; step two, recording of fatal stroke in the community; and step 3, recording of non-fatal strokes that occur in the community.

In 2023, the WSO-Lancet commission on stroke recommended national stroke registers as a research priority, and in 2022, the African Stroke Leaders Summit prioritized the establishment of stroke registers as a leading research priority for the continent.^23^ This scoping review aims to map where stroke registers have been implemented in SSA. We chose to study registers in SSA, as opposed to all African countries, to maintain alignment with global health and demographic data sources and as previous analyses in the stroke literature have used these regional classifications. The article then compares and critiques the methods and definitions used and summarizes key results from the registers.

## Methods

The scoping review is structured according to the Joanna Briggs Institute (JBI) framework “Guidance for conducting systematic scoping reviews.”^24^ Due to the broad definition of what might be considered a register, we adopted broad search terms. We used a definition of a stroke register as “a prospective stroke cohort study which is a by-product of routine clinical care.”^25^

This study does not report any individual-level patient data and only summarizes previously published research findings; therefore, we did not seek ethical approval.

We searched EMBASE, MEDLINE, and CABI Global Health databases. A full list of search terms and full search strategy is included in the Supplemental Material. We included publications from the year 2005, the date when the WHO Stepwise approach to stroke surveillance was published, until the date of search, 8 December 2023. We conducted backward snowballing by checking reference lists of articles included after our first screening and compared records found against our inclusion and exclusion criteria.^26^ We then conducted forward snowballing of all included studies. We included all studies with a prospective longitudinal design in SSA, where acute stroke among adults was the primary condition studied with at least 50 participants, as preliminary searches found many low-quality studies with small sample sizes. We excluded retrospective studies; cross-sectional studies; stand-alone studies which were unrelated to patient care (such as household surveys or door-to-door prevalence studies); studies where stroke was not the primary condition; studies outside of SSA; studies focused on radiological diagnostic accuracy; studies focused on pediatric patients; and studies written in a language other than English.

Articles were screened against inclusion and exclusion criteria independently by two authors DY and MB. Any disagreements on inclusion were rectified in a meeting between the two reviewers, through consensus. No decisions needed to be elevated to a third reviewer, IJM. As the objective was to describe stroke registers, as opposed to individual publications, data from multiple publications concerning one register were aggregated into the description of one register. Geographical location of the registers is presented in map format using MapChart.

Data were extracted from the retrieved publications in the following manner. Publications were assessed as to whether they referred to themselves as “registers” or “registry” in the text of their publication, then all cohorts that met our definition of a register were included. Publications were scored against the MDR-OK criteria (Mergeable data, Dataset standardized, Rules for data collection, Observations over time, Knowledge of outcomes). The methods section and references were searched to identify the use of the WHO Standard method for implementing stroke registers.^22^ Publications were searched for the case definition used in case ascertainment and diagnosis and were classified as the WHO International Statistical Classification of Diseases Tenth Revision (ICD-10) definition^27^ or other. Classification of subtype of ischemic stroke was categorized as Oxfordshire Community Stroke Project (OCSP),^28^ Trial of ORG 10172 (TOAST),^29^ or anatomical (WHO definition). Stroke severity scales were categorized as National Institutes of Health Stroke Scale (NIHSS),^30^ Glasgow Coma Scale (GCS), none, or other. Functional measures were categorized as Barthel Index (BI),^31^ modified Rankin Scale (mRS),^32^ other, or none. Health-related quality-of-life (HRQoL) measures are described by instrument name. Publications were searched for information reported on care seeking and pre-hospital care and were classified as including care-seeking information if they reported information on time to seek care, distance from hospital, or treatment prior to care or transport method of arrival. Publications were classified as including quality-of-care information if they described hyperacute stroke care or medications prescribed or swallowing assessment or urinary catheter use or rehabilitation. The use of socioeconomic measures (educational attainment, socioeconomic measure, income or assets of participants) was also documented. We documented funding sources from the publications and categorized them as local, national, or international. Electronic versus paper-based data collection was recorded alongside the database software used.

The results are tabulated and described below. Count, percentage, mean, and median are presented for numerical variables as per the original source manuscript, and summary statistics are presented at the register/study level, as opposed to the participant level.

## Results

**Figure 1.**
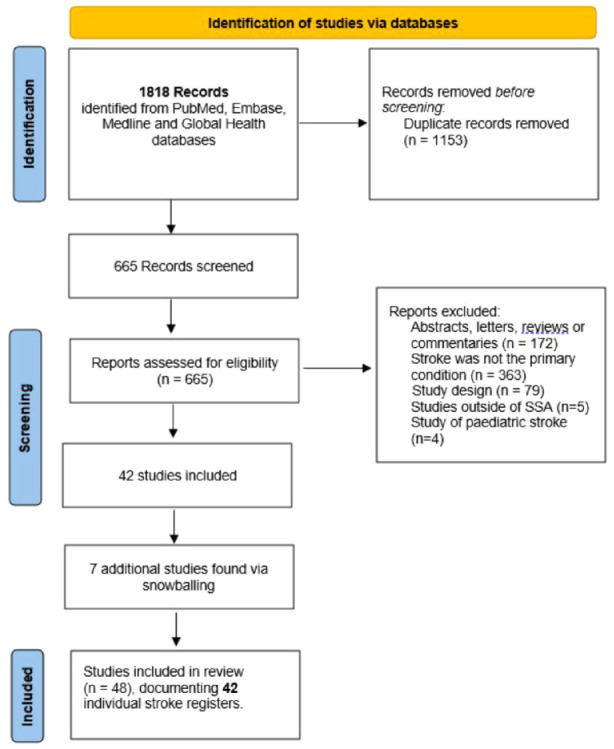
PRISMA flowchart of study identification, screening, and inclusion.

**Figure 2.**
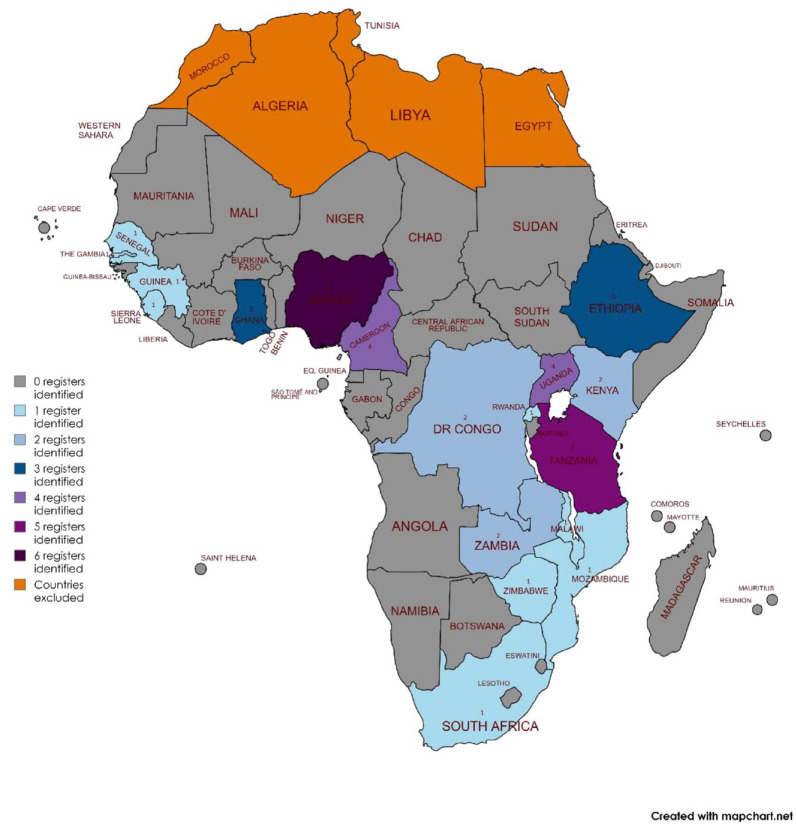
Geographical distribution of stroke registers in Sub-Saharan Africa.

We identified 42 unique stroke registers from 48 individual studies, see Figure 1. The registers were located in 19 countries, with 19 from East Africa, 15 West Africa, 6 Central Africa, and 2 from Southern Africa (Table 1). Their geographic distribution is shown in Figure 2. Cumulatively, the registers recruited 12,345 participants with stroke, the median number of participants was 183 (IQR: 121–312), and the range was 50–1018.

**Table 1.** Number of stroke registers, size, key results, and methodology used by region.

	No. of registers	Mean participants	Age mean (SD)	Percentage male	Percentage ischemic	Percentage neuroimaging	Use of term register, %	Use of WHO definition of stroke, %	Includes NIHSS, %	Includes functional measure, %
Central	6	262	59.5 (3.4)	51.3%	69%	85%	17%	67%	50%	33%
Eastern	19	274	59.2 (5.8)	51.6%	61%	86%	21%	74%	53%	68%
Southern	2	362	59.9 (9.4)	58.5%	62%	91%	50%	0%	100%	100%
Western	15	323	59.4 (3.6)	54.0%	60%	80%	20%	53%	60%	60%
Total	42	293	59.4	53%	62%	84%	21%	26%	57%	62%

### Register design

Only one study was a population-based register, and 41 were hospital-based registers; full details of individual registers are shown in Supplementary material Table 1. Of the hospital-based registers, 29 were single site, 10 were conducted at two sites, and 2 at three sites (see Table 1). Forty of 42 registers were recruited at the tertiary hospital level, and 23 were located in the capital city of their respective country. Length of recruitment ranged from 4 months to 6 years, and the median length of recruitment was 12 months. The median number of participants recruited per month was 20 and ranged from 1 to 59. The majority of registers recruited patients with incident and recurrent stroke, and one register in Burkina Faso focused solely on recurrent stroke. Follow-up periods varied, 17 registers followed up to in-hospital outcomes only, 13 registers included a 28-day or 1-month follow-up, four registers had a 90-day follow-up, five included a 1-year follow-up, and the population-based register followed up until 7–10 years after stroke.

Only six (16.6%) of the cohorts referred to themselves as stroke registers. Seven (19.4%) registers referenced the WHO STEPwise approach to implementing stroke registers. The MDR-OK criteria score ranged from 4 to 6. All registers scored 1 on the “mergeable data,” 1 on “dataset standardized,” 1 on “rules for data collection,” and 1 on “observations over time” criteria. Registers dropped points on the “knowledge of outcomes” criteria as some registers did not report outcomes, thereby scoring 0 or 1 out of the possible two points.

### Measures and definitions of registers

Twenty-seven (64.3%) registers used the WHO definition of stroke, see Supplementary Material Table 1. The mean neuroimaging rate was 84% and ranged from 0 to 100%; 21 (50%) had a 100% rate of neuroimaging and used it as an inclusion criteria for the register. Twenty-four registers did not use a classification system to subtype ischemic strokes, seven registers used OCSP, four registers used anatomical site, and three registers used TOAST criteria. Stroke severity was measured using the NIHSS in 22 (52.4%) registers, 4 registers used GCS, 2 registers used the miniNIHSS, 1 used the Scandinavian Stroke Scale, 1 mRS, and 11 registers did not report a stroke severity measure. Seventeen (40.5%) registers used the mRS to measure function, six registers used the BI alone, and three registers used both mRS and BI. Only two registers included a quality-of-life measure, the EQ-5D. Thirteen registers recorded information on pre-hospital care seeking. Eight registers included a quality-of-care measure, including antiplatelet prescription, urinary catheter use, swallowing assessment, or care processes such as time to see physician.

### Sociodemographic information

The proportion of patients who were male ranged from 33% to 73%; the median was 53%. The mean age at stroke occurrence was 59 years, ranging from 53 to 69 years. Twenty-six (61.9%) registers recorded socioeconomic status (SES) or a SES proxy, and the most frequently used one was educational attainment in 14 (33.3%) registers.

### Stroke subtypes and outcomes

All registers except one focusing on ischemic stroke only included all stroke types, the proportion of ischemic strokes ranged from 100% to 36.5%, and the median was 55%. Eleven registers reported NIHSS, and the median NIHSS was 11 (range: 9–20; see Table 1). Sixteen registers reported the rate of pneumonia in their cohort, with a median of 23% and a range of 9%–34%. Six registers reported prevalence of urinary tract infections, ranging from 8% to 29%. Twelve registers reported prevalence of urinary tract infections, ranging from 15% to 44%. Thirty-day mortality ranged from 14% to 57%. Five registers reported mortality at 90 days, with a median of 37% and a range of 23%–43%. Eleven registers reported 1-year mortality, ranging from 26% to 76%.

Results of registers aggregated by region are shown in Table 1. Disaggregated results are shown in the Supplementary Material Table 1.

Nineteen registers did not declare a funding source, 16 registers were supported by international funding, 4 registers by local funding sources, and 3 by national funding sources. The most frequent limitations reported by authors were selection bias due to being hospital-based studies and unavailability or access barriers to neuroimaging.

## Discussion

The registers were distributed across East, West, Central, and Southern Africa. The majority of registers (61%) were located in the capital city of the study country. Only the population-based study and one hospital-based register, in Kenya, was located in a self-described rural setting, with the remainder being located in other urban settings. The Kenya rural register had the highest mean age, 69 years, and the lowest proportion of male participants, 38%, older than the register in urban Kenya with a mean age of 62 years and 56% of male participants. The only population-based register found age-standardized incidence rates to be higher in the urban cohort than those in the rural cohort. Fifty-eight percent of the SSA population live in rural areas,^33^ with evidence that risk factors^34^ and barriers to care differ. Stroke registers in rural areas, although harder to establish with less access to neuroimaging^35^ and trained research teams, are a research gap.

In our review, all stroke registers except one were hospital-based, fulfilling only the first step of the WHO Stepwise surveillance for stroke, thereby not providing data on fatal and non-fatal strokes in the community who do not access hospital care. The registers have been used to describe the population at risk and identify associated risk factors, providing relevant local data to healthcare workers and policy-makers. However, excluding one population-based study, all registers in our review were hospital-based; due to selection bias, they are likely not to be representative of the general population; and differences in case-mix make comparing data between registers difficult. No registers in our review had been able to progress to step 2, identification of fatal stroke in the community, and step 3, identification of non-fatal stroke in the community, of the WHO stepwise surveillance for stroke method, to provide representative population-level data. There was less evidence of registers being used to monitor trends over time or for long-term population surveillance of chronic noncommunicable diseases. There was little evidence of registers being used to evaluate or monitor interventions, with the significant exception of stroke register data being used to evaluate the effect of stroke unit–based care in Guinea.^36^

The MDR-OK score ranged from 4 to 6 in the included registers, and as per the criteria, it may be that some of the included registers are not “true” clinical registries; however, some registers which scored 4 were self-described as stroke registers. Registers that scored less than six were deducted points due to not describing outcomes or only describing in-hospital outcomes.

Methodology and definitions were heterogenous between the registers, and using a standardized clinical definition of stroke and a common stroke severity scale would allow for greater comparison between registers and adjustment for case-mix. Twenty (47.6%) of the registers used neuroimaging as an inclusion criterion, and this may introduce selection bias, with patients who are unable to pay for neuroimaging or those too sick to transfer for neuroimaging or who die before neuroimaging being excluded. Most registers measured functional status after stroke, using either mRS or the BI, and few studies mentioned whether they translated or culturally adapted these measures. There was a near absence of HRQoL measures, most HRQoL studies in SSA have been cross-sectional,^37^ and incorporating HRQoL measures into registers provided an important patient-reported outcome measure, but also can be combined with survival data to estimate quality-adjusted life years (QALYs). A majority of registers collected a SES measure, most commonly educational attainment; however, few studies presented results disaggregated by SES. In other settings, stroke registers have been instrumental in measuring inequalities in stroke, and the shared use of educational attainment by registers in our review may enable future meta-analyses in SSA. In the registers with follow-up beyond the initial admission, there was considerable out-of-hospital mortality, and registers that only collect in-hospital outcomes may significantly underestimate stroke mortality. The WHO method includes a 28-day follow-up, reasoning that there will be reduced loss to follow-up; however, patients may still be admitted, and 90-day follow-up may be more appropriate and/or is a better surrogate for patient status at 1 year.

While individual studies presented heterogenous results, results aggregated by region provided similar and consistent results, a mean age of 59 years, percentage of male sex at 53%, and an ischemic stroke proportion of 62%. There was greater heterogeneity in CFR after stroke, noted by others,^38^ as these were unadjusted for stroke severity (with average NIHSS ranging from 9 to 21 in our review). The inclusion of NIHSS in the majority 54.8% of the registers in our review offers the potential for future analysis of CFR in SSA adjusted for or stratified by stroke severity.

### Recommendations

Standardization in both the definition of stroke and its data collection is needed to ensure the quality, usability, and comparability of stroke registers. For stroke registers in SSA, we recommend using the WHO clinical definition of stroke and the WHO Stepwise method of stroke surveillance tool with some additional information. Including time of stroke onset and admission to hospital in addition to date would allow more specific data on hours from stroke onset to admission to be collected and pave the way for hyperacute stroke care. Expansion of quality-of-care indicators to include early mobilization, catheter usage, and the collection of urinary tract infections and seizures as complications may be advisable. Follow-up timepoints may be changed from 30 to 90 days to improve evaluation of longer-term survival and functional status and to ensure an out-of-hospital timepoint is captured as some patients may still be admitted at 30 days. As part of the expanded data collection, the inclusion of a patient reported outcome measure (PROM), such as the EQ-5D which yields QALYs for use in future economic evaluations, would help monitor patient-centered care. Access to neuroimaging was a frequently reported limitation by authors of the registers, with studies either using this as an exclusion criterion or removing patients with stroke of unknown type from their analysis. This is important to address, as these patients may suffer severe strokes and die early in admission or be unable to pay for neuroimaging. To provide more accurate and comparable data, we recommend the inclusion of these patients as stroke of unknown type. Finally, our review clearly shows the lack of population-based registers and registers collecting stroke data at primary and secondary levels and in rural locations, which may hide inequities in stroke occurrence, care, and outcomes.

Our review is limited by only including English language publications. English is only a formal language in 27 of 46 (58.7%) SSA countries. The cutoff time period for study inclusion was 2005, the year of publication of the WHO Stepwise method for stroke surveillance, and we therefore do not focus on stroke registers established before this. We were unable to contact all study authors and therefore could not assess whether any registers were ongoing or clarify if multiple studies came from the same register. Our definition of a stroke register as “a prospective stroke cohort study which is a by-product of routine clinical care” is by necessity broad and may have included studies whose design does not aim or aspire to be a registry. Simultaneously we excluded studies with similar aims but of different designs such as the SIREN^39,40^ and INTERSTROKE^41^ studies.

Our scoping review is timely, exploring the use of stroke registers in SSA, where stroke incidence is increasing and evidence is sparse, at a time when stroke registers are being highlighted internationally and regionally as a research priority. We highlight two research gaps, rural populations in SSA and population-based research, which stroke registers could address. We describe heterogeneity in methodology used, recommend areas of standardization, and their utility for future research and highlight useful additions such as the inclusion of HRQoL measures.

## Supplemental Material

sj-xlsx-1-wso-10.1177_17474930241262936 – Supplemental material for A scoping review of stroke registers in Sub-Saharan Africa Supplemental material, sj-xlsx-1-wso-10.1177_17474930241262936 for A scoping review of stroke registers in Sub-Saharan Africa by Daniel Youkee, Mamadu Baldeh, Anthony Rudd, Marina Soley-Bori, Charles DA Wolfe, Gibrilla F Deen and Iain J Marshall in International Journal of Stroke
